# The Role of Glucocorticoid Receptor and Oxytocin Receptor in the Septic Heart in a Clinically Relevant, Resuscitated Porcine Model With Underlying Atherosclerosis

**DOI:** 10.3389/fendo.2020.00299

**Published:** 2020-05-14

**Authors:** Tamara Merz, Nicole Denoix, Daniela Wigger, Christiane Waller, Martin Wepler, Sabine Vettorazzi, Jan Tuckermann, Peter Radermacher, Oscar McCook

**Affiliations:** ^1^Ulm University Medical Center, Institute for Anesthesiological Pathophysiology and Process Engineering, Ulm, Germany; ^2^Clinic for Psychosomatic Medicine and Psychotherapy, Ulm University Medical Center, Ulm, Germany; ^3^Department of Psychosomatic Medicine and Psychotherapy, Nuremberg General Hospital, Paracelsus Medical University, Nuremberg, Germany; ^4^Clinic for Anesthesia, Ulm University Medical Center, Ulm, Germany; ^5^Institute of Comparative Molecular Endocrinology, Ulm University, Ulm, Germany

**Keywords:** sepsis, heart, oxytocin receptor, glucocorticoid receptor, cystathionine-γ-lyase, oxidative stress, inflammation

## Abstract

The pathophysiology of sepsis-induced myocardial dysfunction is not resolved to date and comprises inflammation, barrier dysfunction and oxidative stress. Disease-associated reduction of tissue cystathionine-γ-lyase (CSE) expression, an endogenous H_2_S-producing enzyme, is associated with oxidative stress, barrier dysfunction and organ injury. CSE-mediated cardio-protection has been suggested to be related the upregulation of oxytocin receptor (OTR). CSE can also mediate glucocorticoid receptor (GR) signaling, which is important for normal heart function. A sepsis-related loss of cardiac CSE expression associated with impaired organ function has been reported previously. The aim of this current *post hoc* study was to investigate the role of cardiac GR and OTR after polymicrobial sepsis in a clinically relevant, resuscitated, atherosclerotic porcine model. Anesthetized and instrumented FBM (Familial Hypercholesterolemia Bretoncelles Meishan) pigs with high fat diet-induced atherosclerosis underwent poly-microbial septic shock (*n* = 8) or sham procedure (*n* = 5), and subsequently received intensive care therapy with fluid and noradrenaline administration for 24 h. Cardiac protein expression and mRNA levels were analyzed. Systemic troponin, a marker of cardiac injury, was significantly increased in septic animals in contrast to sham, whereas OTR and GR expression in septic hearts were reduced, along with a down-regulation of anti-inflammatory GR target genes and the antioxidant transcription factor NRF2. These results suggest a potential interplay between GR, CSE, and OTR in sepsis-mediated oxidative stress, inflammation and cardiac dysfunction.

## Introduction

Myocardial dysfunction is present in 20–70% of patients with sepsis and often complicated by coronary artery disease (CAD), which has a significant impact on mortality (1–4). The pathophysiology of septic cardiomyopathy is not fully understood and includes inflammation, barrier dysfunction and oxidative stress (5). The lack of cystathionine-γ-lyase (CSE) expression, an endogenous H_2_S producing enzyme, is associated with oxidative stress and barrier dysfunction, as previously reported in sepsis-induced acute kidney injury (6, 7).

CSE and endogenously produced H_2_S have cardioprotective effects in heart failure (8). Septic cardiomyopathy is associated with reduced cardiac and cardiovascular CSE expression in a co-morbid pig model (9, 10). OTR expression in the heart is directly affected by H_2_S (11, 12). Recently, the reperfusion injury salvage kinase (RISK) pathway was suggested to regulate CSE-mediated cardio-protection by increasing OTR expression (13). Interestingly, H_2_S is also implicated in the hypothalamic regulation of heart rate and blood pressure by stimulating OT release during fluid shifts (14). Sepsis is characterized by intravascular fluid shifts and vasodilation (15). Oxytocin receptor (OTR) signaling, in turn, is also critical for heart function, has vasodilatory effects and regulates blood pressure and body fluid homeostasis (16). However, little is known about its role in sepsis.

Glucocorticoids have been implicated in the regulation of oxytocin (OT) synthesis and secretion in response to altered fluid volume and tonicity (17). Glucocorticoid receptor (GR) signaling is important for normal heart function and development (18). Moreover, we have recently demonstrated that impaired GR dimerization aggravated hemodynamic instability and organ dysfunction during LPS-induced circulatory shock (19). Results for GR expression in peripheral blood cells in sepsis are ambivalent (20, 21). While a GR down-regulation is associated with organ dysfunction in the septic liver (22), there are no reports of cardiac tissue expression during sepsis.

Finally, impaired cardiac H_2_S/OTR signaling is associated with cardiac injury in a murine model of psychological trauma (12) and in a murine acute on chronic injury model, and OTR was restored by exogenous H_2_S administration (11). CSE is a regulator of GC signaling (23) and critical for appropriate GC production in the adrenal gland during sepsis (24). Therefore, the aim of this *post hoc* study was to investigate the expression of GR and OTR in order to elucidate their putative role in the heart after sepsis in a clinically relevant, resuscitated, atherosclerotic large animal model.

## Materials and Methods

The study was approved by the University of Ulm Animal Care Committee and the Federal Authorities for Animal Research. The experiments were performed in adherence to the National Institute of Health Guidelines on the Use of Laboratory Animals and the European Union “Directive 2010/63/EU on the protection of animals used for scientific purposes” and authorized by the federal authorities for animal research of the Regierungspräsidium Tübingen (approved animal experimentation number: 1024), Baden-Württemberg, Germany, and the Animal Care Committee of the University of Ulm, Baden-Württemberg, Germany. This is a *post hoc* study performed on available material from the vehicle-treated group of a previous study (25) and sham-operated animals studied simultaneously under the same protocol (7, 9). The underlying atherosclerosis in the pig strain has previously been characterized in the coronary vasculature by our group (9, 10).

### Experimental Protocol and Measurements

Briefly, male castrated FBM (Familial Hypercholesterolemia Bretoncelles Meishan) pigs [age 15–30 months, 69 kg (65–73 kg)] with a high-fat diet-induced hypercholesterolemia and atherosclerosis (26) underwent polymicrobial septic shock (*n* = 8) induced by inoculation of autologous feces into the abdominal cavity, or sham procedure, i.e., abdominal saline injection (*n* = 5), and subsequently received intensive care therapy for 24 h. Anesthesia and surgical instrumentation have been previously described in detail (25). Notably, the septic and sham pigs had the right jugular vein and left carotid artery exposed for the insertion of a central venous catheter sheath and the placement of a balloon-tipped pulmonary artery catheter to measure central venous pressure (CVP), a thermistor-tipped arterial catheter for blood pressure [mean arterial pressure (MAP)] recording and transpulmonary single indicator thermodilution–cardiac output measurement and placement of a left-ventricular catheter for the assessment of left-ventricular function (9, 25). Animals were allowed to recover for 12 h before the induction of sepsis. Mean arterial pressure was maintained at baseline target values by the continuous infusion of Ringer's solution, hydroxyethyl starch infusion and administration of noradrenaline based on need (25). Noradrenaline infusion was not further increased, if the heart rate was higher than 170/min to avoid tachycardia-induced myocardial ischemia. The physiological data obtained in this model were all published previously (7, 9, 10) and are summarized in the Table S1. Sepsis was confirmed by the presence of hyperlactatemia (>2 mmol/l) and significant hypotension in spite of adequate fluid and catecholamine resuscitation in the septic animals (see Table S1). Furthermore, septic animals had left-ventricular dysfunction in that the stroke volume and ejection fraction could only be maintained by noradrenaline administration, concomitant with disturbed diastolic relaxation, as evidenced by the unchanged left-ventricular end-diastolic volume even though the pulmonary artery occlusion pressure was even higher in sepsis [Table S1, (9)]. Twenty-four hours after the induction of fecal peritonitis, anesthesia was further deepened and animals were sacrificed with potassium chloride (7). In addition to the previously published assessment of cardiac function (9), in the current study plasma troponin was determined as a marker of cardiac injury at baseline and 24 h after sepsis.

### Immunohistochemistry

Post-mortem, left-ventricular cardiac samples were fixed in formalin, dehydrated, and embedded in paraffin blocks. Immunohistochemistry was performed as described previously (7, 9, 10). Paraffin sections (3–5 μm) were cut, deparaffinized in xylene, and rehydrated with a graded series of ethanol to deionized water. After heat-induced antigen retrieval in citrate pH 6, the slides were blocked with 10% normal goat serum (Jackson ImmunoResearch) before incubating in primary antibody [1° ab, anti-GR (D8H2, cell signaling), and anti-PGC1α (Novus)]. Primary antibody detection was performed by Dako REAL detection system (anti-mouse, anti-rabbit; alkaline phosphatase conjugated) and visualized with red chromogen (Dako REAL; Dako) followed by counterstaining with hematoxylin (Sigma). The slides were visualized using a Zeiss Axio Imager A1 microscope with a × 10 objective. Quantification for intensity was performed on multiple 800,000-μm^2^ sections using the AxioVision 4.8 software (Zeiss) (7). Data are presented as densitometric sum red.

### Quantitative Polymerase Chain Reaction

Left-ventricular cardiac samples were snap frozen in liquid nitrogen immediately post mortem and stored at −80°C. RNA was extracted by homogenization with tissue homogenisator (Precellys®) and Trizol (invitrogen) following the manufacturer's instructions. RNA quality was checked using the nanodrop (thermofisher). DNaseI treated RNA (1 μg) was used to generate cDNA by oligo(dT) priming. qRT-PCR was performed with the ViiA™ 7 Realtime PCR System (Life technologies) using a Platinum SYBR Green (Invitrogen) and analyzed with the QuantStudio Realtime-PCR software using the ΔΔCτ method. β-Actin served as housekeeping gene. The pig specific primers for the analyzed target genes were obtained from Sigma with the sequences listed in Table 1.

**Table 1:** Primer sequences for mRNA analysis.

**Gene**	**Forward primer**	**Reverse primer**
Actin	ctaggagcgggttgaggtg	ctggtctcaagtcagtgtacaggt
GILZ	atcagctgcacaatttcaaca	tccagcttaacggaaaccac
ATF4	gggctgaagagagcttaggg	acccatgaggtttgaagtgc
VEGFA	atcttcaagccgtcctgtgt	acactccagaccttcgtcgt
HIF1α	aggaacctgatgctttaactttgt	tgtgtcattgctgccaaaat
PPARG1a	gtgaccactgagaatgaggcta	ggctcttctgcctcctga
NRF2	ggtttcttcggctacatttca	agcctggttaggagcaatga
CEBP	tgtgtacagatgaatgataaactctgc	gattgcatcaacttcgaaacc
PPARg	catgctgtcatgggtgaaac	cagacagcgtgtcgaagg
IL10	cacatgctccgggaactc	ggtccttcgtttgaaagaaactc
Dusp	cccgttgaggacaaccac	tgaaatcgattgcctcattg
CSE	tccaccacgttcaaacaaga	ttccagaacggctgtactca
SphK1	cgcctcttctcgacctca	ctgctctcacccgaccac
FKBP5	agacccgggactggtgac	ccctggcaccctctaagc
ZFP36	tcaccagtttcactgccttg	agggaggcaggagtatggaa

*GILZ, glucocorticoid-induced leucine zipper; ATF4, activating transcription factor 4; VEGFA, vascular endothelial growth factor A; HIF1α, hypoxia inducible factor 1α; PPARG1a, peroxisome proliferator-activated receptor gamma coactivator 1-alpha; NRF2, nuclear factor erythroid 2-related factor 2; CEBP, CCAAT-enhancer-binding proteins; PPARg, peroxisome proliferator-activated receptor gamma; IL10, interleukin 10; Dusp, dual-specificity phosphatase; CSE, cystathionine-γ-lyase; SphK1, sphingosine kinase 1; FKBP5, FK506 (tacrolimus) binding protein 5; ZFP36, zinc finger protein 36 homolog (or tristetraprolin TTP)*.

### Statistical Analysis

Statistical analysis was performed with GraphPad Prism Version 4. Data are presented as median (quartiles) or single plotted values with median and interquartile range. Troponin data were analyzed with a two-way ANOVA and *post hoc* Tukey test for multiple comparisons. All other inter-group differences were analyzed with the Mann–Whitney rank sum test after exclusion of normal distribution using the Kolmogorov–Smirnov test. Analysis of mRNA data was performed after excluding outliers according to Grubbs' test.

## Results

**Figure 1:**
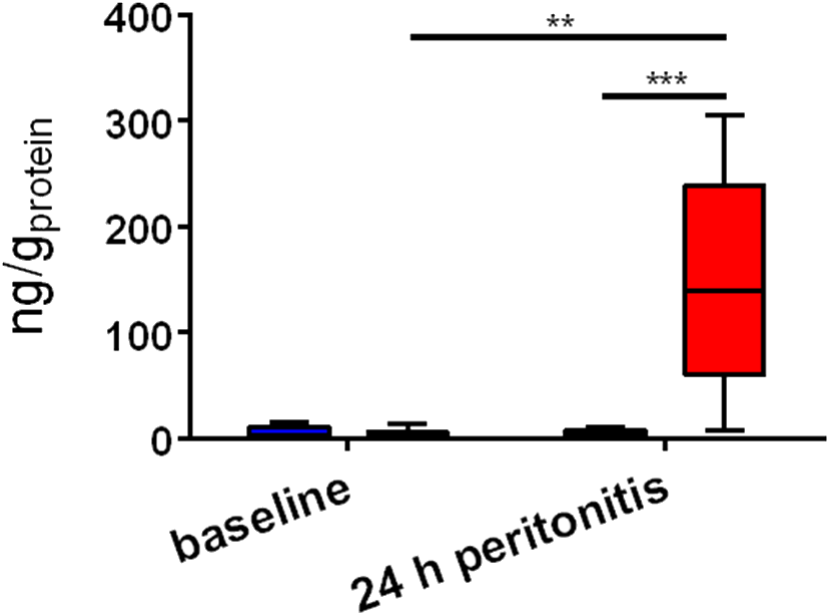
Systemic troponin levels. The x-axis indicates the timepoint of measurement, whereas the y-axis displays plasma troponin levels in ng/g_protein_. The blue box (left box for each timepoint) represents sham (*n* = 5), the red box (right box for each timepoint) represents sepsis (*n* = 8). **p<0.01; ***p<0.001.

**Figure 2:**
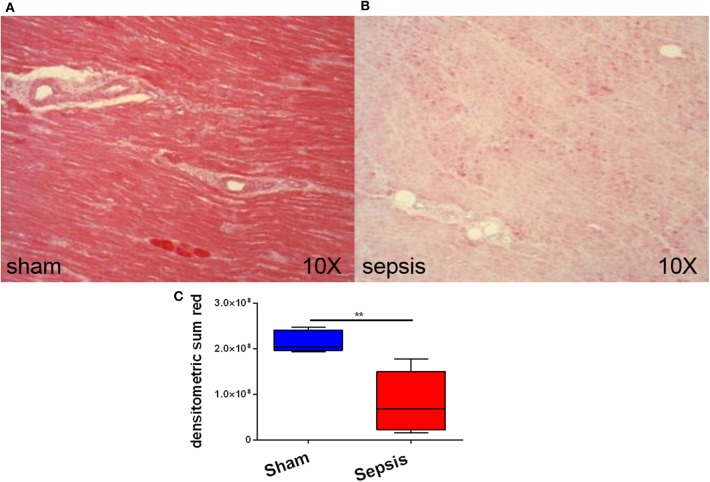
Myocardial oxytocin receptor (OTR) expression. **(A)** shows an example of immunohistochemical staining of OTR in a sham animal and **(B)** shows an example of immunohistochemical staining of OTR in a septic animal. **(C)** displays the quantification of the immunohistochemical stainings as densitometric sum (red), sham: *n* = 5, sepsis: *n* = 8. Boxes represent the interquartile ranges with the median indicated by a black line, whiskers represent minimum and maximum values. **p<0.01.

**Figure 3:**
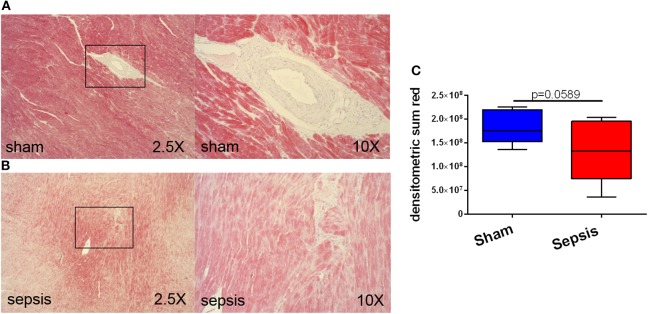
Myocardial glucocorticoid receptor (GR) expression. **(A)** shows examples of immunohistochemical staining of GR in a sham animal at 2.5X and a higher magnification of an arteriole. **(B)** shows examples of immunohistochemical staining of GR in a septic animal in two different magnifications. The black boxes indicate the location where the higher magnification picture was taken. **(C)** displays the quantification of the immunohistochemical stainings as densitometric sum (red), sham: *n* = 5, sepsis: *n* = 8. Boxes represent the interquartile ranges with the median indicated by a black line, whiskers represent minimum and maximum values.

**Figure 4:**
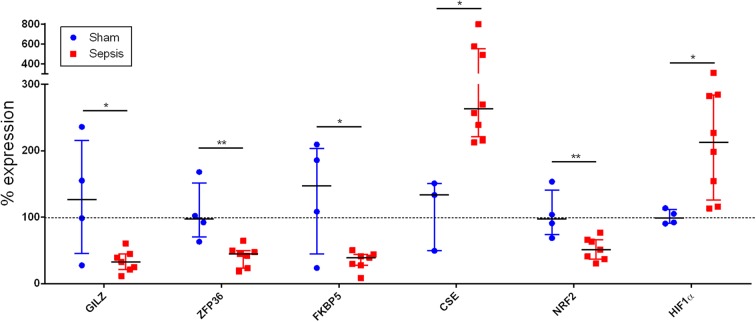
Cardiac mRNA expression. The x-axis lists the analyzed genes, whereas the y-axis indicates % expression normalized to sham. GILZ, glucocorticoid-induced leucine zipper, ZFP36: zinc finger protein 36 homolog (or tristetraprolin TTP), FKBP5: FK506 (tacrolimus) binding protein 5, CSE, cystathionine-γ-lyase, NRF2, nuclear factor erythroid 2-related factor 2, HIF1α, hypoxia inducible factor 1α. sham: *n* = 4, sepsis: *n* = 8. *p<0.05; **p<0.01.

At 24 h after sepsis induction, systemic troponin levels, as a marker of myocardial injury, were increased 37-fold in septic animals in contrast to sham (*p* = 0.009) (see Figure 1). Immunohistochemistry revealed the presence of the OTR protein and its expression in septic hearts was significantly reduced in comparison to sham animals (*p* = 0.001) (see Figure 2). GR protein was also expressed in the heart and reduced in sepsis (*p* = 0.059) (see Figure 3). Cardiac mRNA expression levels of GR target genes (*Dusp, SphK1, IL10, GILZ, ZFP36*), genes related to H_2_S (*ATF4, VEGF, NRF2, CSE*) and genes related to both H_2_S and GR (*CEBP, PGC1a, PPARg, HIF1*α) were quantified. *Dusp, SphK1, IL10, ATF4, VEGF, CEBP, PGC1a*, and *PPARg* were not significantly affected by sepsis (data not shown). Levels of *GILZ* (*p* = 0.018), *ZFP36* (*p* = 0.006), *FKBP5* (*p* = 0.012) and *NRF2* (*p* = 0.009) mRNA were reduced, whereas *CSE* (*p* = 0.012) and *HIF1*α (*p* = 0.020) were elevated in sepsis (see Figure 4).

## Discussion

In this clinically relevant, co-morbid, resuscitated large animal model of sepsis, we show (i) expression of OTR and GR in the porcine heart, (ii) a sepsis-induced loss of cardiac OTR and GR expression, coinciding with (iii) increased systemic troponin levels as a marker of cardiac injury, (iv) impaired GR signaling reflected in low levels of GR target genes, (v) increased levels of CSE and HIF1α mRNA, and (vi) a lower level of NRF2 mRNA, suggesting an impaired antioxidant defense.

Given the fact that OT/OTR and GC/GR are involved in the regulation of fluid balance and vascular tone, the goal of this study was to further investigate their role and regulation in the heart of sepsis-induced hypotension. In an effort to increase the translational impact of pre-clinical studies in sepsis research, we chose to investigate atherosclerotic pigs (FBM) with a similar biomarker profile to septic patients with CAD: significantly higher cholesterol levels, increased oxidative stress and lower blood levels of nitric oxide (NO) metabolites (7). As was reported by Raper & Sibbald, patients suffering from coronary artery disease (CAD) present with a lower cardiac output in response to sepsis than otherwise healthy patients (1). In contrast to young healthy pigs, the FBM pigs display a similar reduction of cardiac output as patients with CAD in response to sepsis (10). Moreover, during resuscitation from hemorrhagic shock, this pig strain requires significantly higher noradrenaline doses to achieve similar hemodynamic targets (27, 28). Aggravated septic cardiomyopathy can be related to atherosclerosis, chronic kidney disease and cardiac dysfunction, all of which are associated with reduced tissue CSE expression [coronary artery (10), kidney (7), heart (9)].

In the present study elevated troponin levels confirm cardiac injury in the septic arm (Figure 1), supporting the findings that increased CSE mRNA expression (Figure 4) is an up-regulation in compensation for the loss of cardiac CSE protein expression (9). In line with these results was the loss of OTR in the septic hearts (Figure 2), since CSE-mediated cardio-protection is suggested to work through the up-regulation of OTR via the RISK pathway (13). In fact, in CSE knock-out mice trauma led to a reduction of cardiac OTR expression and the exogenous administration of H_2_S led to higher levels of OTR compared to the vehicle group (11). Both OT and H_2_S have been reported to be involved in NRF2 signaling, which is an important antioxidant transcriptional regulator (29–31). This is confirmed in the present study: low levels of NRF2 and OTR (Figures 2, 4) taken together with low levels of CSE protein coincided with high nitrotyrosine, a marker of oxidative and nitrosative stress (9). NRF2 converges with H_2_S/OT in the RISK pathway, mediating cardio-protective effects through endothelial NO synthase and the subsequent production of NO (13, 29, 30). However, under conditions of oxidative stress, NO can react with superoxide and generates peroxynitrite, resulting in nitrotyrosine formation by the nitration of protein tyrosine residues (9, 32). Thus, nitrotyrosine formation is a sign of injury, reflective of reduced NO bioavailability and impaired NO signaling (9, 32), as also reflected in the coronary arteries of septic atherosclerotic pigs (10). Oxidative stress can induce the transcription of HIF1α (33), which in turn can upregulate the GR (34). The upregulation of HIF1α in the present study (Figure 4) suggests that it is trying to compensate for the low levels of GR protein expression (Figure 3). This is the first report of sepsis-related organ dysfunction in relation to GR expression in the heart. The GR reduction is in agreement with Jenniskens et al., who reported sepsis-induced organ dysfunction related to a down-regulation of GR in the liver (22). This is in contrast to the ambivalent reports of GR expression in circulating cells (20, 21), which may not necessarily represent the role of GR at the organ level during sepsis (35). As reviewed by Cavaillon and Annane (35), in sepsis, organ specific gene expression was determined to share common patterns or show distinctly opposite profiles between different organs (36), whereas circulating cytokines were not reflective of tissue-specific local levels (37). These differences in pathophysiological events between organs and systemic factors during sepsis led to the concept of compartmentalization (35), thus the focus in this study was on an organ-specific effect of sepsis.

Anti-inflammatory GR signaling is crucial for survival in several animal models of sepsis (38–40) and related to the suppression of TNFα-induced inflammation (39). Of the known investigated (anti-inflammatory) GR dependent mediators, only FKBP5, ZFP36, and GILZ were statistically significant in the septic arm of the present study (Figure 4). FKBP5 expression is known to be induced by GC-signaling (41), thus the reduced levels (Figure 4) are a sign of reduced GR activity. The reduction of ZFP36 (Figure 4) was associated with elevated levels of TNFα (7), in support of data from the literature (42). The lower anti-inflammatory GILZ expression as a consequence of low GR activity in sepsis is also confirmed in the literature: low levels of GILZ have been detected in septic patients (43, 44). Thus, the dysregulation of ZFP36 and GILZ both confirm impaired GC anti-inflammatory signaling in the septic arm.

### Limitations

Decreased cardiac contractility in sepsis can be mediated by reduced activity of L-type calcium channels (45). A lack of GR is associated with low levels of L-type calcium channels and related left-ventricular dysfunction (46) and L-type calcium channels also play an important role in OT-induced muscular contraction (13). Thus, L-type calcium channels would have been a potentially interesting downstream target of GR and OTR in the context of this study, which has not yet been investigated. Another limitation of this study is the fact, that septic animals received significantly more noradrenaline based on the need to maintain the mean arterial pressure in comparison to sham animals, thus it cannot be excluded that the observed cardiac injury and protein dysregulation might be due to noradrenaline administration rather than sepsis. Even though OT can reportedly affect endogenous catecholamine release and -responsiveness (47, 48), nothing is known about the effects of exogenous high-level noradrenaline administration on OT/OTR signaling in the heart. Glucocorticoid signaling can potentiate noradrenaline signaling in various brain regions (49, 50), but there are no reports in the literature about their relationship in the heart.

## Conclusion

In our septic co-morbid pig model, septic cardiomyopathy was associated with reduced CSE, OTR, and GR expression and signaling, oxidative stress, increased troponin levels and systemic inflammation (7, 9). Taken together, these results suggest a potential interplay between GR, CSE, and OTR in sepsis-mediated cardiac dysfunction.

## Data Availability Statement

Datasets are available on request. The raw data supporting the conclusions of this article will be made available by the authors, without undue reservation, to any qualified researcher.

## Ethics Statement

The animal study was reviewed and approved by Federal authorities for animal research of the Regierungspräsidium Tübingen, Baden-Württemberg, Germany.

## Author Contributions

TM contributed to the experimental design, performed experiments, analyzed data, and drafted the manuscript. MW, ND, and DW performed experiments. SV helped with data analysis. JT and CW critically reviewed and edited the manuscript. PR designed the experiment and critically reviewed and edited the manuscript. OM designed the experiment, analyzed data and critically reviewed and edited the manuscript. All authors read and approved the final version of the manuscript.

## Conflict of Interest

The authors declare that the research was conducted in the absence of any commercial or financial relationships that could be construed as a potential conflict of interest.
