# Supplementary material for: The Role of Glucocorticoid Receptor and Oxytocin Receptor in the Septic Heart in a Clinically Relevant, Resuscitated Porcine Model With Underlying Atherosclerosis
Source: Front Endocrinol (Lausanne). 2020 May 14;11:299. doi: 10.3389/fendo.2020.00299 (PMC7239997; doi:10.3389/fendo.2020.00299)
Supplement: Supplementary file 1 [file Table_1.pdf]

Supplemental Material

Table S1: Physiological Parameters, previously published as indicated. Data are given as median (interquartile range)

		baseline	24 h peritonitis
Noradrenaline infusion rate [1] ($\mu g/(kg \cdot min)$	sham	0.06 (0.02; 0.13)	
	sepsis	1.23 (0.66; 3.26)[a]	
Hydroxyethyl starch infusion rate (mL/min)	sham	3 (2; 4)	
	sepsis	10 (10;12)[a]	
Heart rate [2] (L/min)	sham	88 (73; 104)	102 (68; 115)
	sepsis	88 (74; 106)	156 (140; 166)[a,b]
Mean arterial pressure [2] (mmHg)	sham	100 (90; 106)	103 (94; 119)
	sepsis	103 (91; 112)	71 (62; 101) [a,b]
Central venous pressure [2] (mmHg)	sham	8 (7; 13)	10 (9; 17)[b]
	sepsis	10 (6; 13)	17 (14; 18)[b]
Cardiac output [1] ($mL/(kg \cdot min)$)	sham	61 (52; 79)	64 (42; 92)
	sepsis	64 (52; 69)	87 (62; 130)[b]
Stroke volume [2] (mL)	sham	50 (46; 59)	47 (43; 54)
	sepsis	44 (39; 67)	42 (31; 66)
Ejection fraction [2] (%)	sham	45 (44; 49)	39 (34; 49)
	sepsis	50 (41; 61)	60 (40; 78)
Left ventricular end-diastolic volume (LVEDV) [2] (mL)	sham	116 (102; 129)	131 (97; 168)
	sepsis	94 (78; 118)	86 (62; 114)
Pulmonary artery occlusion pressure (PAOP) [2] (mmHg)	sham	9 (7; 16)	11 (7; 16)
	sepsis	11 (6; 13)	18 (16; 20)
Arterial pH [3]	sham	7.46 (7.44; 7.46)	7.44 (7.43; 7.46)
	sepsis	7.45 (7.43; 7.48)	7.37 (7.19; 7.43)[a,b]
Base excess [3]	sham	1.1 (0.8; 1.8)	-0.1(-1.45; 0.65)
	sepsis	1.5 (0.4; 2.3)	-8.5 (-14.6; -3.7) [a,b]
Lactate [3] (mmol/L)	sham	1.4 (1.0; 1.6)	0.6 (0.6; 1.2)
	sepsis	0.8 (0.6; 1.5)	6.1 (2.0; 10.7) [a,b]
Urine output [3] ($ml/(kg \cdot h)$)	sham	n.d.	8 (6;11)
	sepsis	n.d.	4 (2; 5)[a]
Plasma creatinine [3] (µmol/L)	sham	n.d.	89 (79; 92)
	sepsis	n.d.	126 (110; 138)[a]
Creatinine Clearance [3] (mL/min)	sham	n.d.	112 (105; 121)
	sepsis	n.d.	56 (35; 84)[a]
Neutrophil gelatinase associated lipocalin [3] $ng/g_{protein}$)	sham	1.8 (1.6; 1.9)	6.4 (6.4; 6.9)[b]
	sepsis	1.4 (1.1; 2.0)	35.0 (18.8; 56.7)[a,b]

1 previously published in Merz *et al. Intensive Care Medicine Experimental* (2017) 5:17
2 previously published in Nußbaum *et al. Intensive Care Medicine Experimental* (2016) 4:14
3 previously published in Merz *et al. Intensive Care Medicine Experimental* (2018) 6:43

a significant to sham
b significant to baseline
